# AXL is a potential therapeutic target in dedifferentiated and pleomorphic liposarcomas

**DOI:** 10.1186/s12885-015-1916-3

**Published:** 2015-11-14

**Authors:** Caitlin D. May, Jeannine Garnett, XiaoYan Ma, Sharon M. Landers, Davis R. Ingram, Elizabeth G. Demicco, Ghadah A. Al Sannaa, Tona Vu, Lixia Han, Yi Zhang, Christine M. Kivlin, Svetlana Bolshakov, Azad Abul Kalam, Juehui Liu, Fuguo Zhou, Dominique Broccoli, Wei-Lien Wang, Alexander J. Lazar, Raphael E. Pollock, Dina Lev, Keila E. Torres

**Affiliations:** 1 Department of Surgical Oncology, The University of Texas MD Anderson Cancer Center, Houston, TX USA; 2 The University of Texas Health Science Center at Houston Graduate School of Biomedical Sciences, Houston, TX USA; 3 Department of Pathology, The University of Texas MD Anderson Cancer Center, Houston, TX USA; 4 Department of Cancer Biology, The University of Texas MD Anderson Cancer Center, Houston, TX USA; 5 Curtis and Elizabeth Anderson Cancer Institute, Memorial University Medical Center, Savannah, GA USA; 6 Department of Surgery, The Ohio State University, Columbus, OH USA; 7 Department of Surgery, Sheba Medical Center, Tel Aviv University, Tel Aviv, Israel

**Keywords:** AXL, Receptor tyrosine kinase, Liposarcoma, Soft tissue sarcoma

## Abstract

**Background:**

AXL is a well-characterized, protumorigenic receptor tyrosine kinase that is highly expressed and activated in numerous human carcinomas and sarcomas, including aggressive subtypes of liposarcoma. However, the role of AXL in the pathogenesis of well-differentiated (WDLPS), dedifferentiated (DDLPS), and pleomorphic liposarcoma (PLS) has not yet been determined.

**Methods:**

Immunohistochemical analysis of AXL expression was conducted on two tissue microarrays containing patient WDLPS, DDLPS, and PLS samples. A panel of DDLPS and PLS cell lines were interrogated via western blot for AXL expression and activity and by ELISA for growth arrest-specific 6 (GAS6) production. AXL knockdown was achieved by siRNA or shRNA. The effects of AXL knockdown on cell proliferation, migration, and invasion were measured in vitro. In addition, AXL shRNA-containing DDLPS cells were assessed for their tumor-forming capacity in vivo.

**Results:**

In this study, we determined that AXL is expressed in a subset of WDLPS, DDLPS, and PLS patient tumor samples. In addition, AXL and its ligand GAS6 are expressed in a panel of DDLPS and PLS cell lines. We show that the in vitro activation of AXL via stimulation with exogenous GAS6 resulted in a significant increase in cell proliferation, migration, and invasion in DDLPS and PLS cell lines. Transient knockdown of AXL resulted in attenuation of these protumorigenic phenotypes in vitro. Stable AXL knockdown not only decreased migratory and invasive characteristics of DDLPS and PLS cells in vitro but also significantly diminished tumorigenicity of two dedifferentiated liposarcoma xenograft models in vivo.

**Conclusions:**

Our results suggest that AXL signaling contributes to the aggressiveness of DDLPS and PLS, and that AXL is therefore a potential therapeutic target for treatment of these rare, yet devastating tumors.

## Background

Soft tissue sarcomas include over 50 histologically distinct subtypes of mesenchymal malignancies and account for approximately 1 % of all adult solid cancers in the United States [1]. Liposarcomas (LPS) are one of the most common subtypes of soft tissue sarcoma, comprising nearly 20 % of cases. The World Health Organization recognizes four distinct histological subtypes of LPS: atypical lipomatous tumor (ALT)/well differentiated LPS (WDLPS), dedifferentiated LPS (DDLPS), myxoid LPS, and pleomorphic LPS (PLS); the tumors in these groups differ in morphology, genetic composition, and clinical behavior [2].

Approximately 40–45 % of LPS are ALT/WDLPS, which are locally aggressive and rarely metastatic. These tumors are characterized by amplification of the 12q13-15 chromosomal region, which ultimately results in the formation of supernumerary rings and/or giant rod chromosomes [3]. In this study, we will use the term WDLPS instead of ALT/WDLPS. WDLPS tumors have a propensity for local recurrence as well as dedifferentiation with subsequent metastasis [2]. A dedifferentiated cellular component within WDLPS occurs in approximately 10 % of cases, and these biphasic tumors are classified as DDLPS [2]. Like WDLPS, DDLPS occurs most frequently in the retroperitoneum and to a lesser extent in the extremities. Nearly all DDLPS cases (90 %) are identified within the primary tumor, while the remainder of incidences may appear in the context of a WDLPS recurrence [2]. DDLPS are typically considered to be intermediate-to-high-grade malignancies, with a local recurrence rate of approximately 60 % and a metastatic rate of 15–20 %, resulting in 5-year survival rates of approximately 30–55 % [2, 4]. PLS, which accounts for only 5 % of LPS diagnoses, is more aggressive, exhibits complex karyotypes, and tends to develop in deep soft tissues of the extremities, with rare instances of retroperitoneal occurrence [2]. The aggressiveness of PLS is highlighted by a 30–50 % metastatic rate, a five-year survival rate of 25–60 %, and an overall survival rate of 40–50 % [2, 5, 6]. Currently, there is a lack of effective chemotherapeutic or targeted therapies for WDLPS, DDLPS, and PLS, with surgical resection remaining the primary standard of care for patients with these sarcomas [7]. Biological insights are needed to identify molecular mechanisms that may be clinically useful as potential therapeutic targets in LPS.

We recently showed that AXL was highly upregulated in LPS cells versus normal preadipocytes and adipocytes [8]. AXL is a member of the TYRO3-AXL-MER (TAM) family of receptor tyrosine kinases (RTKs). Activation of AXL via its ligand GAS6 (growth arrest-specific 6) stimulation promotes cellular processes such as survival, proliferation, migration, and cell-cell adhesion, while inhibiting apoptosis [9–13]. In addition, overexpression of AXL has been implicated in the development and progression of numerous malignancies; furthermore, AXL has been identified as a predictor of poor patient outcome in lung, breast, and pancreatic cancer, renal cell carcinoma, and glioblastoma [9, 14–23]. High levels of activated AXL have been linked to increased chemoresistance in lung and breast cancers, esophageal carcinoma, and acute myeloid leukemia [24–27].

It has also been reported that RNAi-mediated knockdown of AXL in pancreatic adenocarcinoma and breast and lung cancer cells decreases migration, invasion, tumor growth, and metastasis and also impairs angiogenesis [18, 19, 28–31]. Taken together, those previous studies suggest that AXL may be a key signaling node regulating several biological processes during tumorigenesis and cancer progression. However, the role of AXL in the pathogenesis of LPS has not been determined. Our study aimed to investigate the role of AXL in WDLPS, DDLPS, and PLS through the analysis of patient samples, cell lines, and xenograft models.

## Methods

### Clinically annotated tissue microarray

This study was approved by The University of Texas MD Anderson Cancer Center (UTMDACC) institutional review board and informed consent was obtained from all patients. By using the UTMDACC prospective sarcoma database, institutional tumor registry, and pathology archives, 46 WDLPS and 19 DDLPS formalin-fixed paraffin-embedded samples from 45 patients who presented to UTMDACC from January 2000 to February 2009 and subsequently underwent surgical resection were selected for tissue microarray (TMA) inclusion. The TMA was constructed as previously described, with two 0.6-mm punches per sample embedded into a TMA block [32]. Hematoxylin-eosin verification was performed on 4 μm sections. An additional TMA composed of 39 PLS patient samples from 32 patients that was previously constructed by our research group was also used to examine AXL expression [33]. Normal fat (NF; *n* = 12) specimens were included as a control.

### Cell lines

Human DDLPS cell lines Lipo-224A, Lipo-224B, Lipo-246, and Lipo-863, the DDLPS cell strain Lipo-573, and the PLS cell line PLS-1 were derived and cultured in our laboratory as previously described [8]. Lipo-573 was subsequently removed from our panel due to observed senescence. The PLS cell lines LS2 and LiSa2 were maintained as previously described [34]. The DDLPS cell line LPS141 was kindly provided by Dr. Jonathan Fletcher (Dana-Farber Cancer Institute, Boston, MA) [35]. Primary human white preadipocytes were purchased from PromoCell (Heidelberg, Germany) and maintained in commercially available preadipocyte growth medium (PromoCell). Human white preadipocytes were differentiated into adipocytes in culture with a commercially available preadipocyte differentiation medium according to the manufacturer’s instructions (PromoCell). Both preadipocytes and adipocytes were used as experimental controls. DDLPS and PLS cells were grown in Dulbecco modified Eagle medium (DMEM) supplemented with 10 % fetal bovine serum (FBS), 100 U/mL penicillin, and 100 μg/mL streptomycin. Established cell lines were validated via short tandem repeat DNA fingerprinting as previously described [36].

### Antibodies and reagents

Commercially available antibodies were purchased for immunoblotting or immunohistochemical detection of AXL. pAXL Y702, AKT, pAKT S473, ERK, and pERK T202/T204 (Cell Signaling Technology, Danvers, MA); MER (GeneTex, Irvine, CA); TYRO3 (Abcam, Cambridge, MA); Ki67 and cleaved caspase 3 (Biocare Medical, Concord, CA); and β-actin–horseradish peroxidase (Santa Cruz Biotechnology, Dallas, TX). Recombinant human GAS6 was purchased from R&D Systems (Minneapolis, MN).

### Western blot analysis

Standard protocols for western blot analyses of whole cell extracts were followed [8]. For GAS6 stimulation experiments, cells were serum-starved in 1%FBS-DMEM overnight and then stimulated for 30 min with 400 ng/mL recombinant GAS6, a concentration previously described to activate AXL in vitro [11, 20, 21].

### Quantification of GAS6 via Enzyme-Linked Immunosorbent Assay (ELISA)

Antibody sandwich ELISAs (R&D Systems, Minneapolis, MN) were used to measure secreted GAS6 in human primary preadipocytes, adipocytes, and DDLPS and PLS cell lines in low-serum conditions (1 % FBS; 24 h). Samples of conditioned DMEM (1 % FBS) were obtained from preadipocytes, adipocytes, and cell lines after 24 h, after which the total number of cells was calculated. Secreted GAS6 levels were normalized to cell number.

### Assessment of cell proliferation

Cell proliferation was evaluated using a CellTiter 96 AQueous One Solution Cell Proliferation Assay (containing 3-(4,5-dimethylthiazol-2-yl)-5-(3-carboxymethoxyphenyl)-2-(4-sulfophenyl)-2H-tetrazolium, inner salt; MTS) (Promega, Madison, WI) according to the manufacturer’s instructions. Briefly, cells were seeded in 96-well plates at a density of 1.5 × 10^3^ cells/well in 100 μL of medium and assayed for proliferation after 48 h. For experiments with GAS6, 24 h after seeding, the medium was replaced with low-serum medium (1 % FBS) with or without 400 ng/mL recombinant GAS6. Cell proliferation was assayed 48 h later (72 h total). For experiments with small interfering RNA (siRNA), cells were seeded 48 h post-transfection and proliferation was measured 48 h thereafter (96 h after transfection). Following the addition of MTS reagent, plates were incubated for 2 h at 37 °C, and absorbance was read at 490 nm. For each experiment, proliferation was assayed in triplicate, and the percent cell proliferation was normalized to the control.

### Modified Boyden chamber assays

Migration and invasion assays were performed using cell culture inserts with 8 μm polyethylene terephthalate (PET) filters with or without Matrigel (Becton Dickinson Labware, Franklin Lakes, NJ). Briefly, 2.5 × 10^4^ cells were seeded in the inserts of modified Boyden chambers in low-serum (1 % FBS) DMEM/F12 medium. DMEM/F12 medium containing 5 % FBS was used as a chemoattractant for these studies. Triplicate wells were used for each experiment. For studies evaluating the effect of GAS6-mediated AXL activation on migration and invasion, 100 ng/mL GAS6 was added within the inserts at the time of cell seeding. For experiments with siRNA, cells were seeded 48 h post-transfection. After 14 h incubation at 37 °C, cells were fixed with 10 % glutaraldehyde and stained with 0.2 % crystal violet in 20 % methanol (Baxter Healthcare, Houston, TX). Migratory and invasive cells were quantified using ImageJ version 1.47 software (http://rsbweb.nih.gov/ij/index.html) [37].

### Transient and stable knockdown of AXL

For transient knockdown of AXL, 20 nM pools of ON-TARGETplus human AXL-targeting siRNA or a 20 nM pool of control nontargeting (NT) constructs (Dharmacon, Lafayette, CO) were introduced into cells using X-tremeGene (Roche, Indianapolis, IN) per the manufacturer’s instructions. Cells were transfected at 45–50 % confluency in six-well plates and seeded for MTS, migration, and invasion assays 48 h after transfection. Knockdowns were evaluated via western blot analyses of whole cell extracts harvested 72 h after transfection.

Stable knockdown of AXL was achieved by transfecting cell lines with the short hairpin RNA (shRNA) pGIPZ lentiviral constructs containing anti-AXL or NT sequences (Open Biosystems, Lafayette, CO; AXL shRNA #1, V3LHS_329651; AXL shRNA #2, V3LHS_329652; NT sequence, RHS4346). Lentiviruses were generated at the MD Anderson shRNA and ORFeome Core. Target cells were infected with lentivirus, followed by selection with 1 μg/mL puromycin. AXL knockdown was confirmed via western blot analyses.

### In vivo experiments

All animal procedures were approved by the UTMDACC Institutional Animal Care and Use Committee. Animals received humane care in accordance with the Animal Welfare Act and the National Institutes of Health Guide for the Care and Use of Laboratory Animals. To evaluate the effect of AXL knockdown on tumorigenicity, suspensions of Lipo-246 or Lipo-863 cells (2.5 × 10^6^ or 3.5 × 10^6^ cells/0.1 mL of phosphate buffered saline (PBS) per mouse, respectively) expressing either AXL-specific or NT shRNA lentiviral constructs were subcutaneously injected into the flanks of 6-week-old female hairless severe combined immunodeficiency (SCID) mice (n = 8 per construct) (Charles River Laboratory, Wilmington, MA). Tumor size was measured biweekly. When control tumors reached a volume of 1500 mm^3^, all mice were sacrificed and their tumors resected, weighed, and frozen or fixed in formalin. Formalin-fixed tissues were subsequently embedded in paraffin for IHC analyses.

### Immunohistochemistry

For TMA and xenograft analyses, AXL immunostaining was independently scored by two observers (E.G.D. and A.J.L.). Tumor cores missing from the TMA sectioned slides were omitted from analyses. Staining was classified as either negative (intensity of stain scored as 0 or 1) or positive (intensity of stain scored as 2 or 3). Xenograft-derived specimens were further analyzed for Ki67 (cell proliferation marker) and cleaved caspase 3 (CC3; apoptosis marker) expression.

### Statistical analysis

In vitro assays were repeated at least three times; the mean and standard error of the mean (SEM) were calculated for each assay. Two-tailed Student *t*-tests were used to assess significance. Means and SEMs were also calculated for xenograft studies. Differences in xenograft volumes and weights between AXL-specific and NT constructs were assessed using a two-tailed Student *t*-test (* = *p* < 0.05 or *** = *p* < 0.001).

## Results

### AXL and its ligand GAS6 are highly expressed in a subset of DDLPS and PLS

**Fig. 1:**
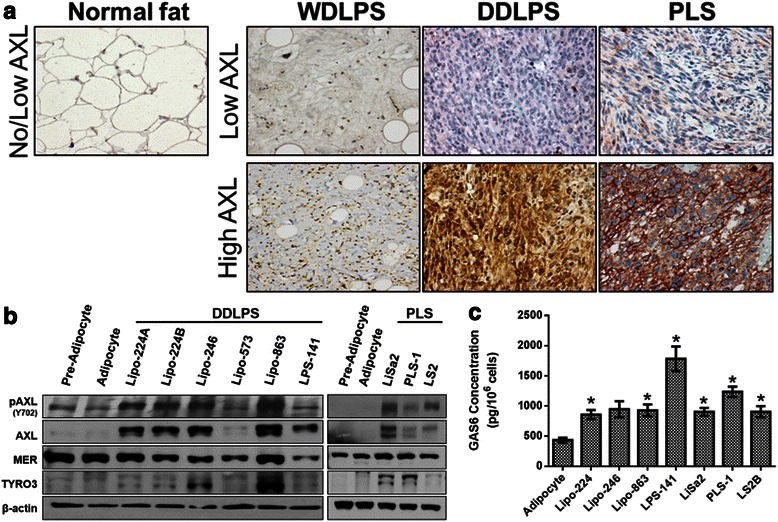
AXL and its ligand, GAS6, are highly expressed in DDLPS and PLS. **a** AXL expression was assessed by IHC analysis of TMAs containing NF samples and WDLPS, DDLPS, and PLS tumor samples (representative images are shown, 200× magnification). **b** Western blot analyses of TAM RTKs in panels of DDLPS and PLS cell lines. **c** GAS6 secretion was measured by ELISA in conditioned media for the indicated DDLPS and PLS cell lines. The data are means with SEMs for triplicate experiments (* = *p* < 0.05)

We first assessed AXL expression in human tumors by conducting IHC analyses on two TMAs containing samples from WDLPS, DDLPS, and PLS patients. AXL expression was observed in 27.7 % (27/104 samples; 23/83 [27.7 %] of patients) of all evaluable LPS samples, with no staining in NF specimens (Fig. 1a). AXL was detected in 14/39 (35.9 %) of PLS samples, 4/19 (21.1 %) of DDLPS samples, and 7/46 (15.2 %) of WDLPS samples (Table 1). The vast majority of WDLPS and DDLPS tumor samples were obtained from tumors in the retroperitoneum, while most PLS samples represented tumors that originated in the extremities. We found that all of the AXL-positive WDLPS and DDLPS samples were obtained from retroperitoneal lesions. In addition, all positive PLS samples were acquired from tumors located in the extremities.

**Table 1:** Summary of AXL immunostaining in patient LPS samples

Histology	Patients (*n*)	Samples^c^ (*n*)	Positive for AXL immunostain	
			Patients (%) (*n* = 83)	Samples (%) (*n* = 104)
NF	12	12	0 (0)	0 (0)
LPS (Total)	83	104	23 (27.7)	27 (26.0)
LPS subtype				
WDLPS				
Extremity	3	3	-	-
Retroperitoneum	31	43	7 (22.6)	7 (16.3)
Total	34	46	7 (20.6)	7 (15.2)
DDLPS				
Extremity	1	1	-	-
Retroperitoneum	17	18	4 (23.5)	4 (22.2)
Total	18	19	4 (22.2)	4 (21.1)
PLS				
Extremity	23	29	10 (43.5)	11 (37.9)
Retroperitoneum	2	2	-	-
Other ^a,b^	7	8	2 (28.6)	3 (37.5)
Total	31	39	12 (37.5)	14 (35.9)

^a^One patient with PLS had a primary tumor in the extremity as well as a metastatic lesion in the lung which was included on the TMA. Therefore, the total number of PLS patients is 31, not 32

^b^Other locations include lung (*n* = 4 patients, 5 samples) and chest/back (*n* = 3 patients, 3 samples)

^c^Several patients had multiple tumor samples included on the TMA

For the in vitro assessment of AXL expression and function, we utilized several previously established DDLPS and PLS cell strains and cell lines; however, WDLPS cell strains do not grow in culture and therefore could not be experimentally tested. Similar to our IHC analysis, immunoblotting of whole cell lysates from DDLPS and PLS cell lines demonstrated that four out of 6 DDLPS cell lines and cell strains and all three PLS cell lines expressed higher levels of both total AXL and its activated form (pAXL Y702) compared with preadipocyte and adipocyte controls (Fig. 1b). In comparison, other TAM family members were not consistently overexpressed, with comparable MER expression levels detected in the control and test cell lines and variable TYRO3 expression in DDLPS and PLS cells.

Next we assessed the levels of the AXL ligand GAS6 in tumor cell-conditioned media in order to determine the potential for autocrine activation of AXL in LPS oncogenicity. We found significantly elevated levels of secreted GAS6 in six out of the seven DDLPS and PLS cell lines when compared to adipocyte control cells, indicating that autocrine stimulation by GAS6 may play a role in AXL activation (Fig. 1c).

### GAS6-mediated AXL activation promotes cell proliferation, migration, and invasion of DDLPS and PLS cells

**Fig. 2:**
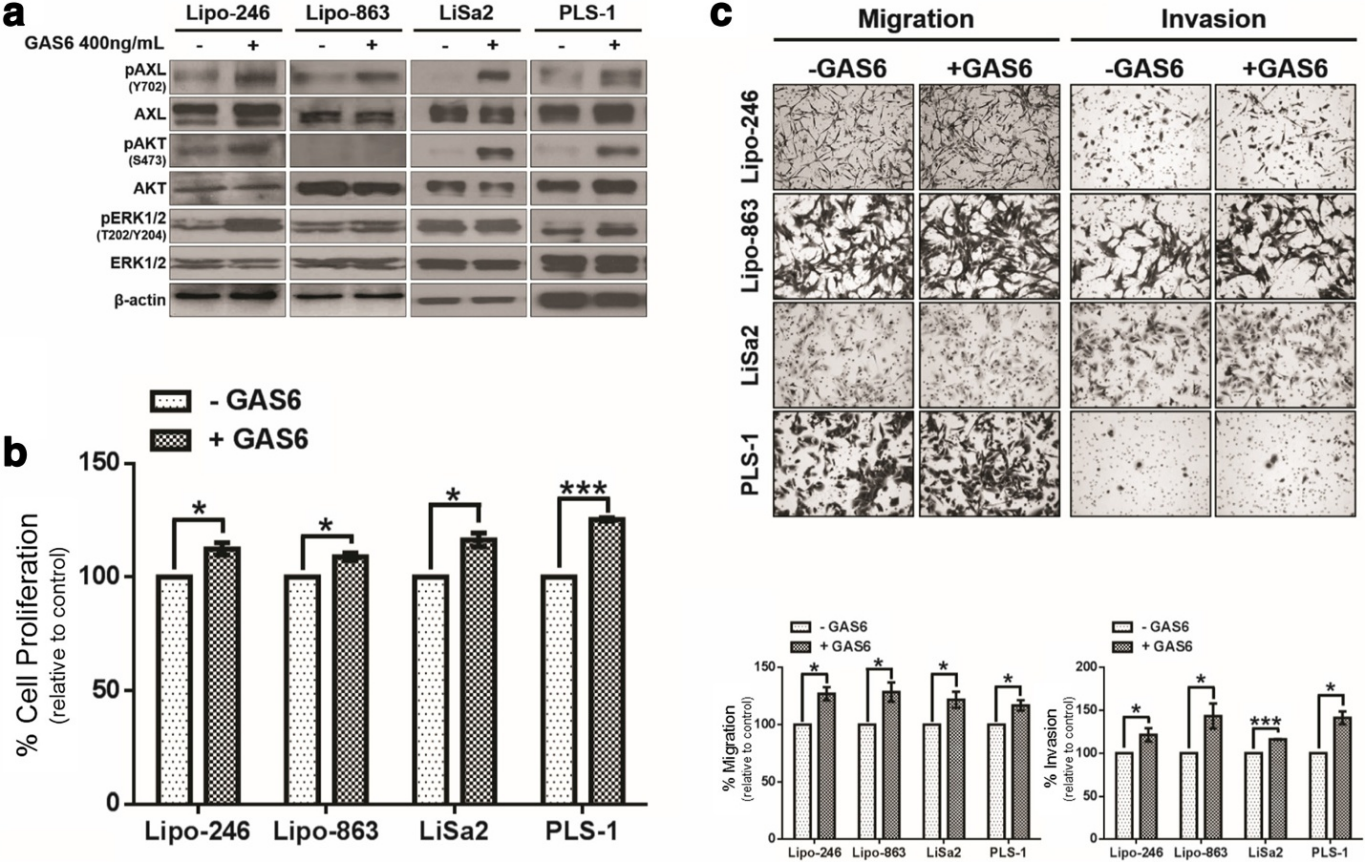
GAS6-mediated AXL activation increases protumorigenic properties of LPS cells in vitro. **a** DDLPS (Lipo-246 and Lipo-863) or PLS (LiSa2 and PLS-1) cells were incubated with low-serum media for 24 h prior to a 15 min stimulation with 400 ng/mL GAS6 and analyzed via western blotting. **b** Changes in DDLPS and PLS cell proliferation were measured by MTS assays with or without GAS6 stimulation for 48 h. Percent of cell growth is expressed as a percentage of control. **c** Modified Boyden chambers were used to assess the effects of GAS6 stimulation on cell migration and invasion. Representative images of each condition are shown (200× magnification). Percent of cell migration and invasion is expressed as a percentage of control. The data in the bar graphs are means with SEMs for triplicate experiments (* = *p* < 0.05; *** = *p* < 0.001)

In order to determine the enhancing effects of GAS6-mediated signaling on the activation of AXL and its downstream effectors in DDLPS and PLS, two DDLPS cell lines (Lipo-246 and Lipo-863) and two PLS cell lines (LiSa2 and PLS-1) were cultured in low-serum conditions overnight, followed by acute exposure to GAS6. Serum starvation did not completely abrogate AXL activation in Lipo-246, Lipo-863, and PLS-1, suggesting that cell line-secreted GAS6 may allow for a baseline level of AXL activation. Exogenous GAS6 stimulation increased AXL activation in all cell lines evaluated; furthermore, Lipo-246, PLS-1, and LiSa2 cells displayed increased phosphatidylinositol-3-kinase (PI3K) pathway activity, as measured by AKT phosphorylation at S473 (Fig. 2a). In most cell lines tested, a minimal increase in the mitogen-activated protein kinase (MAPK) pathway activity was noted, as determined by extracellular signal-regulated kinase (ERK) phosphorylation at T202/Y204. Moreover, the addition of exogenous GAS6 to DDLPS and PLS cells cultured in low-serum media resulted in a moderate, yet significant increase in cell proliferation, cell migration, and invasion (Fig. 2b and c). Taken together, these results indicate that the GAS6/AXL signaling axis enhances protumorigenic phenotypes of DDLPS and PLS cells in vitro.

### Knockdown of AXL decreases protumorigenic properties in vitro

**Fig. 3:**
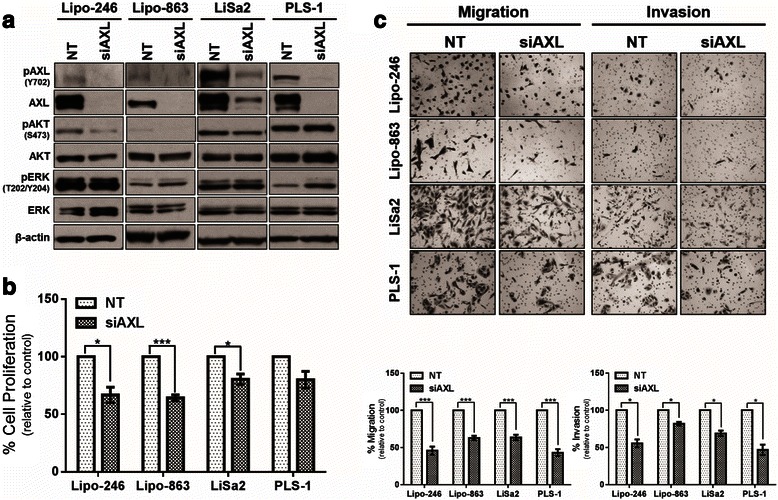
Transient knockdown of AXL decreases cell proliferation, migration, and invasion. **a** Representative western blots showing signaling dysregulation in Lipo-246, Lipo-863, LiSa2, and PLS-1 cells that were transfected with either NT or AXL-specific siRNA. **b** Cell proliferation of AXL-specific siRNA-transfected DDLPS and PLS cell lines was measured by MTS assay after 48 h (96 h post-transfection). Percent of cell growth is expressed as a percentage of control. **c** Modified Boyden chambers were used to assess DDLPS and PLS cell migration and invasion following transfection with AXL-targeting siRNA. Percent of cell migration and invasion is expressed as a percentage of control. The data in the bar graphs are means with SEMs for triplicate experiments (* = *p* < 0.05; *** = *p* < 0.001)

After determining that AXL activation confers proliferative, migratory, and invasive properties to DDLPS and PLS cells in vitro, we wanted to assess whether transient knockdown of AXL could abrogate these processes. Using an anti-AXL siRNA pool, we knocked down AXL in DDLPS and PLS cells; a control pool of four NT constructs was used in parallel. As expected, AXL knockdown resulted in reduced total AXL protein expression and decreased AXL activation (Fig. 3a). We observed a concomitant decrease in AKT activation, suggesting a reduction in PI3K signaling in all cell lines except PLS-1. ERK activity was not strongly affected in any of the cell lines evaluated. Moreover, attenuation of the AXL signaling axis decreased cell proliferation in all cell lines evaluated (Fig. 3b) and markedly reduced the migratory and invasive capabilities of siAXL DDLPS and PLS cells (Fig. 3c).

**Fig. 4:**
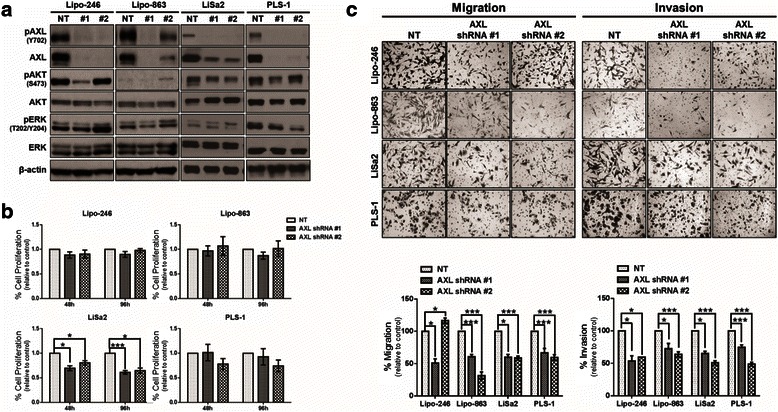
Stable knockdown of AXL in DDLPS and PLS cells reduces cell proliferation, migration, and invasion. **a** Representative western blots showing signaling alterations induced with stable knockdown of AXL in DDLPS and PLS cell lines. **b** MTS assays were performed on DDLPS and PLS cell lines to evaluate cell proliferation after 48 and 96 h in AXL knockdown cells. Percent of cell growth is expressed as a percentage of control. **c** After stable knockdown of AXL, the ability of DDLPS and PLS cell lines to migrate and invade was evaluated. Percent of cell migration and invasion is expressed as a percentage of control. The data in the bar graphs are means with SEMs for triplicate experiments (* = *p* < 0.05; *** = *p* < 0.001)

Next, we stably knocked down AXL to analyze the long-term effects of decreased AXL expression on tumorigenic phenotypes in vitro. DDLPS and PLS cells were transduced either with one of two different AXL-specific shRNA-containing lentiviruses (hereafter referred to as shRNA #1 or #2) or with the NT control construct. Following selection and expansion, western blots were used to examine the degree of stable AXL knockdown in all four cell lines (Fig. 4a). Interestingly, shRNA #1 reduced both AKT and ERK activities in Lipo-246 and Lipo-863 cells, whereas shRNA #2 had the opposite effect. AKT phosphorylation decreased with both AXL-targeting shRNA constructs in the LiSa2 and PLS-1 cell lines; however, ERK activity varied in the PLS cell lines. With the exception of LiSa2 cells, cell proliferation was unaffected in vitro by stable AXL knockdown (Fig. 4b). In contrast, AXL knockdown significantly reduced the migratory and invasive capacities in all four cell lines with the exception of Lipo-246 cells transduced with AXL shRNA #2; these cells had a significant increase in migration (Fig. 4c). Although the phenotypic responses of the individual cell lines to AXL knockdown varied, migration and invasion were compromised in most cell lines, an effect that may decrease tumorigenicity in vivo.

### Stable AXL knockdown diminishes DDLPS tumor growth in vivo

**Fig. 5:**
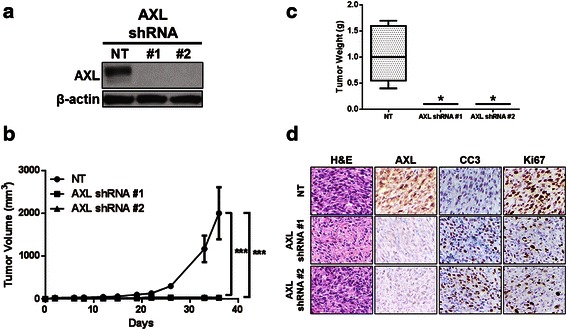
Stable AXL knockdown in Lipo-246 cells significantly reduces tumor weight and volume. **a** AXL knockdown in Lipo-246 cells was evaluated by western blot prior to subcutaneous injection. **b** Tumor volume was monitored at the indicated time points. **c** Xenografts were weighed ex vivo following experiment termination. **d** Representative IHC images of Lipo-246 xenografts expressing NT control constructs or one of two AXL-targeting shRNAs are shown (400× magnification). Hematoxylin-eosin (H&E); cleaved caspase 3 (CC3). (* = *p* < 0.05; *** = *p* < 0.001)

**Fig. 6:**
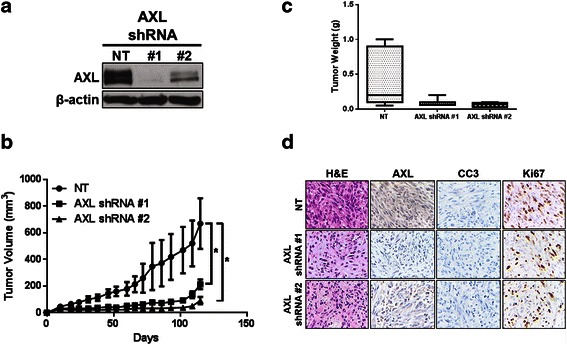
AXL knockdown in Lipo-863 reduces in vivo tumorigenicity. **a** Prior to subcutaneous injection, Lipo-863 cells were assessed for AXL by immunoblotting. **b** Xenograft volume was measured three times weekly. **c** Tumors were harvested and weighed at the end of the experiment. **d** Representative IHC images of Lipo-863 xenografts expressing NT control constructs or AXL-specific shRNAs are presented (400× magnification). Hematoxylin-eosin (H&E); cleaved caspase 3 (CC3). (* = *p* < 0.05)

To evaluate growth of AXL shRNA-transduced DDLPS cells in vivo, we subcutaneously injected Lipo-246 or Lipo-863 cells with or without AXL knockdown (Figs. 5a and 6a) into the flanks of female hairless SCID mice. AXL knockdown in Lipo-246 and Lipo-863 cells significantly reduced tumor volume (*p* < 0.001) compared with that for NT control xenografts (Figs. 5b and 6b, respectively). Additionally, a significant decrease in ex vivo tumor weight (*p* < 0.05) was observed with AXL knockdown in Lipo-246 xenografts; the weight of Lipo-863 tumors with AXL knockdown were decreased compared to the NT control although statistical significance was not achieved (NT v shAXL #1: *p* = 0.055; NT v shAXL #2: *p =* 0.052) (Figs. 5c and 6c). IHC analysis verified decreased AXL expression in shRNA-transduced xenografts (Figs. 5d and 6d). Lower levels of Ki67 expression by IHC confirmed decreased proliferation in both Lipo-246 and Lipo-863 xenografts containing shRNA against AXL compared with NT control xenografts. Lipo-246 AXL knockdown xenografts displayed elevated CC3, suggesting an induction of apoptosis in these tumors.

## Discussion

In this study, we have confirmed that AXL is overexpressed and activated in DDLPS and PLS cell lines and corresponds to enhanced oncogenic signaling through downstream PI3K/AKT and MAPK pathways, which facilitate certain tumorigenic processes such as proliferation, migration, and invasion. While the mechanism of AXL overexpression is outside the scope of this study, we found that DDLPS and PLS cells are capable of producing and secreting GAS6 in an autocrine manner, and that exogenous administration of GAS6 enhances activation of AXL and downstream effectors in these cells. Taken together, these findings support an autocrine mechanism of AXL activation via GAS6 secretion in liposarcoma disease progression. Our observations support previous reports that the role of AXL in cancer is a consequence of receptor overexpression and/or its inappropriate activation [38–40].

AXL signaling is involved in many cellular processes, including survival, migration, invasion, and metastasis in both normal and cancer cells [9–12]. We observed an increase in proliferation, migration, and invasion in our cell line model systems after stimulation with exogenous GAS6, suggesting that activated AXL is an important mediator of these processes in LPS. Most LPS cell lines tested have high endogenous levels of activated AXL in normal growth conditions in vitro; furthermore, Lipo-246, Lipo-863, and PLS-1 had detectable pAXL expression in low serum, which suggests that stimulation via LPS cell-secreted GAS6 may allow for a baseline level of AXL activity and the promotion of proliferation, migration, and invasion. Consequently, increases in these processes after the addition of exogenous GAS6 may be tempered and therefore the observed results are modest but significant.

Initially, we observed that AXL signaling following stimulation with exogenous GAS6 is potentiated primarily through the PI3K/AKT pathway in all cell lines tested, as measured by an increase in pAKT, while a mild increase in signaling through the MAPK pathway was observed in 3 out of 4 cell lines. However, siRNA or shRNA-mediated AXL knockdown did not have a uniform effect on either downstream pathway evaluated. For instance, knockdown of AXL by siRNA resulted in a slight decrease in PI3K/AKT signaling in two cell lines (Lipo-246 and LiSa2), while both pAKT and pERK were elevated in PLS-1. We noted that the effect on downstream pathways were variable following stable AXL knockdown. It should also be noted that Lipo-863 had consistently low or absent basal levels of pAKT and therefore could not be included when assessing pathway preferences for AXL. These data indicate that AXL signaling can be propagated through both the PI3K/AKT and MAPK pathways without any apparent preference, and therefore merits further investigations into alternative downstream pathways.

Our findings for DDLPS and PLS cell lines were similar to those of previous studies in which transient AXL knockdown with siRNA was associated with decreased cell proliferation and a concomitant reduction in migration and invasion [19, 29–31]. Notably, long-term shRNA-mediated knockdown of AXL did not significantly reduce cell proliferation in vitro, which may be due to activation of other pro-tumorigenic RTKs upstream of PI3K or MAPK signaling. We observed that Lipo-246 cells expressing AXL shRNA (specifically, shRNA #2) had high levels of pAKT, supporting the potential for alternative means of pro-LPS pathway activation. Irrespective of downstream signaling, shRNA-mediated AXL knockdown significantly diminished the tumor-forming capacities of Lipo-246 and Lipo-863 cells in vivo, a finding that indicates that AXL may represent a molecular target in a subset of DDLPS tumors. Because reactivation of key canonical downstream pathways was observed in stable AXL knockdown cells in vitro, the possibility exists that tumors may evade AXL blockade in vivo, which could potentially result in acquired resistance to AXL-targeted therapeutics.

The effect of AXL knockdown on in vivo tumorigenic potential is cell line-dependent and may vary with the degree of knockdown. Knockdown of AXL in Lipo-246 cells increased apoptosis and decreased cell proliferation, greatly reducing tumor volume and weight. Lipo-863 shRNA-containing xenografts resulted in decreased cell proliferation, but had minimal effect on apoptosis. These results likely reflect alternate growth factor signaling mediators that are active in different tumors. Knockdown via shRNA #2 did not completely eliminate AXL expression in Lipo-863; however, a significant reduction in tumor volume and weight was noted. These results indicate that moderate knockdown of AXL may be sufficient to reduce tumorigenicity. Additionally in vivo studies interrogating the use of a pharmacological inhibitor against AXL in vivo could further validate our findings and shed more light onto the role of the receptor in disease progression.

Several small-molecule inhibitors of AXL are being investigated as both single agents and in combination for their anti-tumor effects in cancers with high AXL expression [24–27, 41–45]. However, many are multi-target inhibitors known to block signaling through other RTKs such as c-MET, c-KIT, and SRC; therefore, the effects of these inhibitors cannot be fully ascribed to disrupted AXL activity [44, 45]. The AXL-specific inhibitor, R428, has been shown to inhibit cell growth and migration of erlotinib-resistant head and neck cancer cells as well as to resensitize these cells to erlotinib [41]. In our study, we observed examples of potential adaptive resistance in the form of increased PI3K/AKT signaling after shRNA-mediated AXL knockdown in DDLPS cell lines and xenografts, which may render cells insensitive to diminished AXL activity over time. While we have yet to investigate the effects of a small molecule inhibitor in our system, the potential for resistance to reduced AXL expression and activity could indicate the need for combination therapy. Use of both targeted therapy directed at AXL and the PI3K pathway may yield superior anti-LPS effects and improve patient outcome.

AXL has been identified as a predictor of poor prognosis for several types of malignancies, including breast cancer, pancreatic cancer, lung adenocarcinoma, osteosarcoma, oral squamous cell carcinoma, and acute myeloid leukemia [18, 19, 21, 22, 46–48]. To determine whether overexpression of AXL in cultured DDLPS and PLS cell lines is an accurate representation of the human disease, we performed IHC analyses of two TMAs by comparing WDLPS, DDLPS, and PLS patient tumor samples to NF controls. We observed higher levels of AXL expression in DDLPS and PLS patient samples than in NF samples or the lower-grade WDLPS samples. In addition, we focused on the location of all tumors included in the analysis as retroperitoneal WDLPS and DDLPS generally denote a poorer patient prognosis [2]. Interestingly, AXL expression was detected solely in WDLPS and DDLPS that were located in the retroperitoneum. Furthermore, PLS are inherently aggressive tumors regardless of location, and nearly one third of PLS samples were AXL positive. As we did not have comparable numbers of cases in each location, we were unable to make any comparisons between retroperitoneal and extremity WDLPS, DDLPS, and PLS. Additional studies could further validate these findings. However, our results, when combined with previous findings, indicate that AXL may contribute to tumor aggressiveness in DDLPS and PLS and that further studies are warranted to determine the utility of AXL as a potential molecular prognosticator for LPS.

## Conclusion

Overall, our data suggests that AXL is a potential therapeutic target in DDLPS and PLS; further investigations into the effects of AXL-specific targeted therapies for the treatment of LPS are warranted.

## Abbreviations

CC3: cleaved caspase 3
DDLPS: dedifferentiated liposarcoma
DMEM: Dulbecco modified Eagle’s medium
ELISA: enzyme-linked immunosorbent assay
ERK: extracellular signal-regulated kinase
FBS: fetal bovine serum
GAS6: growth arrest-specific 6
H&E: hematoxylin-eosin
IHC: immunohistochemistry
MAPK: mitogen activated kinase
MTS: 3-(4,5-dimethylthiazol-2-yl)-5-(3-carboxymethoxyphenyl)-2-(4-sulfophenyl)-2H-tetrazolium, inner salt
NF: normal fat
NT: nontargeting
PBS: phosphate buffered saline
PI3K: phosphatidylinositol 3-kinase
PLS: pleomorphic liposarcoma
SCID: severe combined immunodeficiency
SEM: standard error of the mean
shRNA: short hairpin RNA
siRNA: small interfering RNA
TAM: TYRO3-AXL-MER
TMA: tissue microarray
WDLPS: well-differentiated liposarcoma
